# Supplementary material for: Prediction of Time Series Gene Expression and Structural Analysis of Gene Regulatory Networks Using Recurrent Neural Networks
Source: Entropy (Basel). 2022 Jan 18;24(2):141. doi: 10.3390/e24020141 (PMC8871363; doi:10.3390/e24020141)
Supplement: Supplementary file 1 [file entropy-24-00141-s001.zip › entropy-1529666-supplementary.pdf]

Prediction of gene expression time series and structural analysis of gene regulatory networks using recurrent neural networks - Supplementary material

Michele Monti [1,2†*], Jonathan Fiorentino [3†], Edoardo Milanetti [3,5], Giorgio Gosti [3] and Gian Gaetano Tartaglia [1,2,3,4*]

[1] RNA System Biology Lab, department of Neuroscience and Brain Technologies, Istituto Italiano di Tecnologia, Via Morego 30, 16163 ,Genoa, Italy.
[2] Centre for Genomic Regulation (CRG), The Barcelona Institute for Science and Technology, Dr. Aiguader 88, 08003 Barcelona
[3] Center for Life Nano- & Neuro-Science, Istituto Italiano di Tecnologia, Viale Regina Elena 291, 00161, Rome, Italy.
[4] Department of Biology and Biotechnology Charles Darwin, Sapienza University of Rome, Rome 00185, Italy.
[5] Department of Physics, Sapienza University of Rome, Rome 00185, Italy.

Correspondence: gian.tartaglia@iit.it; michele.monti@iit.it.

†These authors contributed equally to this work.

This document reports the supplementary material associated to the manuscript ”Prediction of gene expression time series and structural analysis of gene regulatory networks using recurrent neural networks”. We provide additional tables and figures that extend the information contained in the main text. Table S1 reports the parameters used in the Gillespie simulations, Table S2 shows the prediction performance by the DA-RNN for each gene regulatory network. Figure S1 provides an example of the autocorrelation function for a gene belonging to an oscillating gene regulatory network, Figure S2 shows the histograms of the Root Mean Squared Error (RMSE) of the prediction by the DA-RNN for two example gene regulatory networks.

Name	Symbol	Value	Unit
Protein translation rate	$k, \tilde{k}$	0.2	$1/s$
Protein dilution rate	λ	0.01	$1/s$
Hill function exponent	h	2	adim.
Hill function constant	K	50	[a.u.]

Table S1: **Parameter values used in the Gillespie simulations.** We used the same value of the parameters for each gene regulatory network, since the dynamic is driven by the differences in the interaction matrices. The exponent of the Hill function is adimensional, the constant of the Hill function is measured in arbitrary units of concentration.

Network	Predicted data Mean(RMSE)	Std(RMSE)	Input data Mean(RMSE)	Std(RMSE)
FullyConnected	0.97	0.55	0.12	0.04
FullyRepressed	0.86	0.08	0.67	0.08
MasterRegulator	0.94	0.04	0.83	0.04
SparseConnection	0.28	0.15	0.14	0.05
mediumConnection	0.81	0.39	0.35	0.23
Oscillating_nr1_na1	0.49	0.21	0.22	0.11
Oscillating_nr5_na5	0.57	0.32	0.23	0.13
Oscillating_nr10_na10	0.52	0.30	0.30	0.17
Oscillating_nr15_na15	0.20	0.15	0.12	0.09
ExternalSignal_nr1_na1	0.67	0.41	0.29	0.20
ExternalSignal_nr5_na5	0.64	0.35	0.39	0.28
ExternalSignal_nr10_na10	0.16	0.12	0.09	0.06

Table S2: **Prediction accuracy of the DA-RNN for different gene regulatory network architectures.** Network types are those described in section 3.2 of the manuscript, where n_r and n_a indicates the mean number of repressors and activators per gene, respectively. We report the mean and standard deviation of the Root Mean Squared Error (RMSE) computed over all the genes in each network. The time series prediction is performed using a time window T of previously predicted values (left) or of the original data (right).

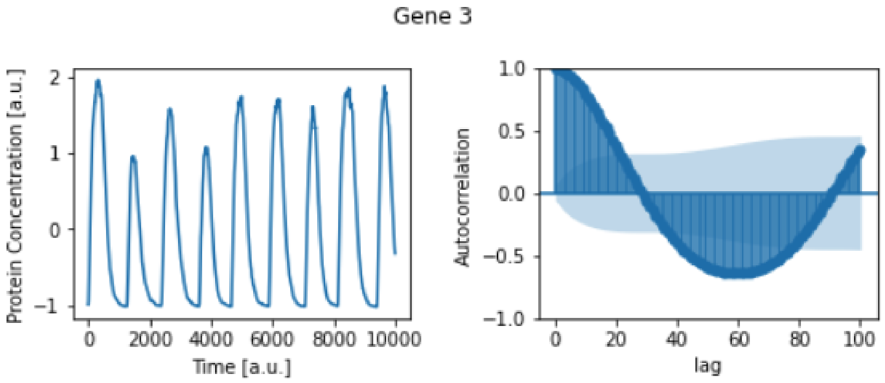

Figure S1: Time series (left) and autocorrelation function (right) for an example gene belonging to an oscillating network (Oscillating_nr10_na10). The shaded area in the right panel represents the 95% confidence interval.

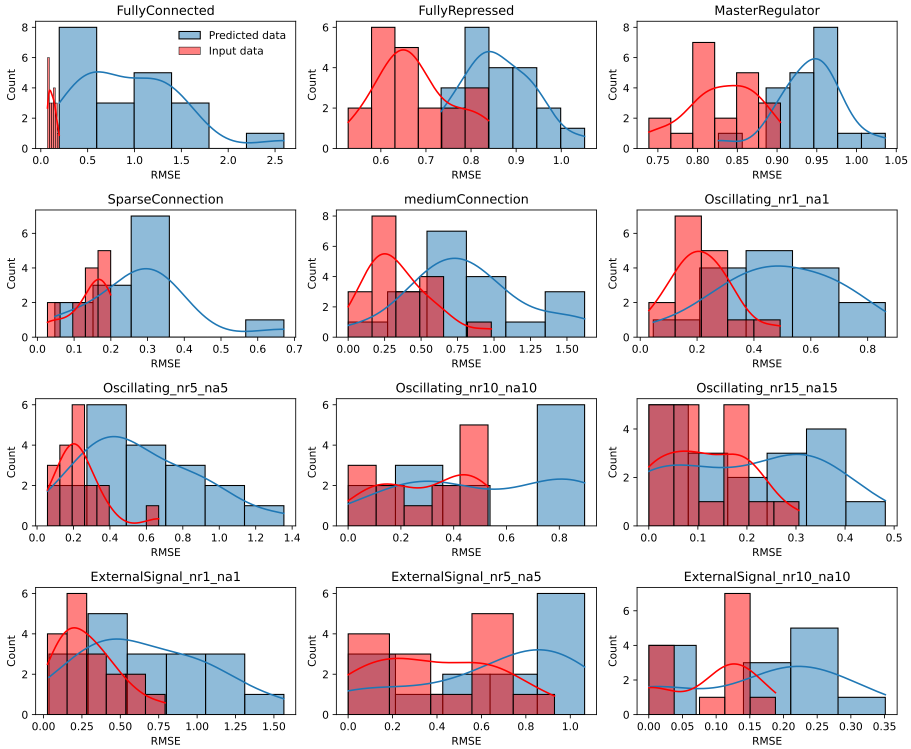

Figure S2: Histograms showing the distribution of the RMSE over all the expressed genes of each gene regulatory network. The time series prediction for RMSE computation is performed using a time window T of previously predicted values (blue) or of the original data (red), as in Supplementary Table 1.
